# “Toxoplasmosis“

**DOI:** 10.1155/S1064744995000019

**Published:** 1995

**Authors:** Sebastian Faro


Infectious Diseases in Obstetrics and Gynecology 2:203–204 (1995)
© 1995 Wiley-Liss, Inc.

### "Toxoplasmosis"

Toxoplasmosis is a disease that has occupied a most curious position in obstetrics for 2 reasons: (1) The lack of an easy, reliable, and economic test and (2) the lack of a safe treatment. Medical students and residents have been taught about this organism and the disease it can cause, more for its academic interest than for its clinical significance. Traditionally, students and residents have been told that they were not likely to see a case of toxoplasmosis. However, with the onset of HIV disease, opportunistic infections have gained clinical significance because of the frequency with which they have begun to occur. Thus, toxoplasmosis has gained prominence as a significant clinical entity, bringing it to the forefront of discussion for the obstetrician.

*Toxoplasma gondii,* a zoonotic parasite, is acquired from infected cats or the ingestion of undercooked meat (beef, pork, or lamb). Transmission can also occur through the transplant of an infected organ or blood or leukocytes. Therefore, the obstetrician must be aware that his or her patient may be susceptible to this infection and that the organism may be acquired through various routes.

Testing for the presence of this infection has significantly improved over the last 5 years. The diagnosis can be established by the detection of *T. gondii,* the presence of oocytes, antigen, antibodies, or nucleic acid. Currently, the most widely used tests are the Sabin-Feldman dye test, complement fixation test, indirect hemagglutination test, indirect fluorescent antibody test, enzyme-linked immunosorbent assay (ELISA), IgM fluorescent antibody test, IgM immunosorbent agglutination assay, and the polymerase chain reaction (PCR) assay.

It is important for the physician to realize that an acute infection in the adult is usually asymptomatic. The symptomatic adult usually presents with posterior cervical lymphadenopathy and an atypical lymphocytosis. The patient may develop malaise, myalgia, headache, fever, skin rash, and splenomegaly. When the acute infection is symptomatic, it is indistinguishable from mononucleosis caused by cytomegalovirus or Epstein-Barr virus.

An intrauterinc infection can occur if the mother experiences a parasitemia, at which time there are circulating tachyzoites. Typically, the mother acquires an infection following the ingestion of oocytes, which penetrate the gastrointestinal mucosa as sporozoites. During the tachyzoite stage, the sporozoites enter the blood stream and gain access to any tissue. The tachyzoite can encyst within a cell and appears to have a preference for striated muscle and brain tissue, remaining there for the life of the host.

In the United States, the incidence of congenital toxoplasmosis is approximately 1 per 1,000 live births. An estimated 40% of women of childbearing age have antibodies to toxoplasmosis, which leaves up to 60% of the women in this age group susceptible to acquiring acute toxoplasmosis.

A congenital infection can result in complications such as mental retardation, seizures, spasticity and palsy, chorioretinitis, glaucoma, optic atrophy, microphthalmia, hydrocephalus, microcephaly, and deafness. The severity of an infection depends upon the trimester in which the infection was acquired. An infection in early pregnancy is less likely to result in intrauterine infection, but

Received January 6, 1995
Accepted January 6, 1995

Editorial more likely to be severe, resulting in abortion, congenital infection with teratogenesis, abnormal growth, or severe residual disease. An infection occurring in the third trimester is likely to result in transplacental transmission, and the congenital infection is likely to be subclinical. These infants usually express their infection later in their development with complications such as learning disabilities or seizures.

Our current ability to document the presence of toxoplasmosis with a high degree of assurance raises the question as to whether all pregnant women should be tested for evidence of past or present *T. gondii* infection. By such testing, we could advise all pregnant women found to be free of antibodies to avoid cats, cat litter, and undercooked meat. However, should these susceptible pregnant women be retested in each subsequent trimester?

*Sebastian Faro, M.D., Ph.D.*
*Editor-in-Chief*

